# Supplementary material for: Kinetic trapping organizes actin filaments within liquid-like protein droplets
Source: Nat Commun. 2024 Apr 11;15:3139. doi: 10.1038/s41467-024-46726-6 (PMC11009352; doi:10.1038/s41467-024-46726-6)
Supplement: Supplementary file 1 — Supplementary Information [file 41467_2024_46726_MOESM1_ESM.pdf]

# Supplementary Materials for

## Kinetic trapping organizes actin filaments within liquid-like protein droplets

Aravind Chandrasekaran[1], Kristin Graham[2], Jeanne C. Stachowiak[2,3*], Padmini Rangamani[1,*]

1. Department of Mechanical and Aerospace Engineering, University of California San Diego, La Jolla, CA 92093-0411
2. Department of Biomedical Engineering, University of Texas at Austin, Austin, TX 78712
3. Department of Chemical Engineering, University of Texas at Austin, Austin, TX 78712

*To whom correspondence should be addressed: jcstach@austin.edu, prangamani@ucsd.edu

# Supplementary Material

**Supplementary Tables**

**Supplementary Table 1. Table of parameters required to set up LAMMPS simulation of VASP-actin system.**

| **Parameter** | **Value** | **Notes/Reference** |
|---|---|---|
| VASP, crowder particle radius | $\sigma = \mathbf{1}$ | |
| **Interaction parameters** | $\epsilon$ - Attraction strength<br>$\sigma$ – distance between centers<br>${\sigma_{LJ}}^{cutoff}$ - cutoff distance | |
| VASP-VASP | 1.66,$\sigma, 2.5\sigma$ | To ensure two-phased system as shown in Figure 3 of Cho et al. [1] |
| Crowder-Crowder | 1.0, $\sigma, 2^{1/6}\sigma$ | |
| Crowder-VASP | 1.0, $\sigma,\ 2^{1/6}\sigma$ | |
| Actin-Actin | 1.0, $0.1\sigma, 1.5\sigma$ | |
| Actin-VASP | 1.2, $0.55\sigma, 2.5\sigma$ | |
| Actin-Crowder | 1.0, $0.55\sigma, 2^{1/6} \times 0.55\sigma$ | Mean radius of Crowder+Actin system |
| Actin harmonic bond potential | Spring constant - $30000 k_B T/\sigma^2$<br>Equilibrium distance - $0.1\sigma$ | Determined empirically to ensure that the contour length of actin does not change within the droplet |
| Actin harmonic angle potential | Energy parameter - 2500$k_B T/rad^2$<br>$k_{bend} = \frac{10394\ pN.nm}{4.11\ pN.nm/k_B T} \sim 2529 k_B T$<br>Equilibrium angle - $180^o$ | Determined from 10394 pN.nm value used by Akenuwa *et al.*[2] |
| **Actin crosslinking parameters** | | |
| Binding sites separation distance | $1.5\sigma$ | Binding sites along the length of an actin filament are spaced apart by this distance |
| Binding distance | $1.2\sigma$ | |

| | | |
|---|---|---|
| Binding event attempted every N steps | 10000 | |
| Binding probability | 0.5 | |
| Maximum number of bonds per site | 1 | |
| Crosslinking bond harmonic potential | Spring constant - $100k_BT/\sigma^2$ Equilibrium distance - $1.1\sigma$ | |

**Supplementary Table 2: Table of parameters required to set up the actin model in Cytosim**

| **Parameter** | **Value** | | **Notes/Reference** |
|---|---|---|---|
| Total time | 600 s | | Experimental images are taken at 600s |
| Implicit evolution time step | 0.002 s | | |
| Viscosity of droplet medium | 0.5 pN/s $\mu m^2$ | | 50x water |
| **Boundary** | | | |
| Shape | Sphere | Ellipsoid | |
| Radius | 1 $\mu m$ | variable | The three axes of the ellipsoid were varied while maintaining the same volume as a sphere. |
| Boundary repulsion stiffness | 200 pN/$\mu m$ for actin filaments and 100pN/$\mu m$ for VASP | | This spring stiffness acts on points if they move outside the boundary and the force depends on the distance outside the boundary. |
| **Actin filaments** | | | |
| Segmentation length, $L_{seg}$ | 100 nm | | |
| Maximum length | 2piR μm | | R is radius of droplet |
| Growing speed | 10.3 nm/s | | Only plus ends are allowed to |

| | | |
|---|---|---|
| | | grow. This rate is calculated to allow actin filaments reach a length of 2piR at 600s |
| Brownian ratchet force for polymerization | 10 pN | [3] |
| Actin flexural rigidity | 0.075 pN $\mu m^2$ | [4] |
| Actin steric repulsion | Radius 3.5 nm<br>Stiffness 1.0 pN/$\mu m$ | Chosen to ensure ring formation is observed in the kinetic parameters explored in Figure 4 |
| Number of filaments | 30 unless mentioned otherwise. | Figures S10 and S11 explore the role of number of filaments. |
| **VASP tetramers** | | |
| Radius | 30 nm | |
| Diffusion rate | 10 $\mu m^2\ /s$ | |
| Concentration of tetramers [VASP-tet] | {0.2,0.4,0.8,1.0,2.0} $\mu M$ | Varied in this study |
| Binding rates | $\{10^{-4},10^{-3},10^{-2},10^{-1},10^{0},10^{+1}\}$ (1/s) | In this study |
| Binding distance | 30 nm | This distance represents the proximity between an actin filament and a VASP molecule required to ensure binding. This value was chosen after a parameter sweep to ensure adequate crosslinking is observed in the simulations. |
| Valency | 4 | Each spherical molecule approximates a tetrameric VASP |
| Unbinding rates | $\{10^{-4},10^{-3},10^{-2},10^{-1},10^{0},10^{+1}\}$ (1/s) | Varied in this study |

| | | |
|---|---|---|
| Characteristic unbinding force | 10 pN | Typical values for passive crosslinkers [5] |
| VASP steric repulsion | Radius 30 nm<br>Stiffness 1.0 pN/$\mu m$ | Chosen to ensure ring formation is observed in the kinetic parameters explored in Figure 4 |

Supplementary Table 3. Table of actin shapes corresponding to each $K_{bind}$ and $K_{unbind}$ value.

| $k_{bind}$ ($s^{-1}$) \ $k_{unbind}$ ($s^{-1}$) | $10^{-4}$ | $10^{-3}$ | $10^{-2}$ | $10^{-1}$ | 1.0 | 10 |
|---|---|---|---|---|---|---|
| 10 | Shell with weak bundling | Shell with weak bundling | Shell with strong bundling | Shell with strong bundling - 33.33%<br>Ring - 66.67% | Ring | Shell with strong bundling |
| 1.0 | Shell with weak bundling | Shell with weak bundling | Shell with strong bundling | Shell with strong bundling - 33.33%<br>Ring - 66.67% | Shell with strong bundling | Shell with strong bundling |
| $10^{-1}$ | Shell with weak bundling | Shell with weak bundling | Shell with strong bundling | Shell with strong bundling | Shell | Shell |
| $10^{-2}$ | Shell with weak bundling | Shell with weak bundling | Shell | Shell | Shell | Shell |
| $10^{-3}$ | Shell | Shell | Shell | Shell | Shell | Shell |
| $10^{-4}$ | Shell | Shell | Shell | Shell | Shell | Shell |

**Supplementary Table 4. Table of parameters used to simulate role of linear nucleator in determining actin organization within droplet**

| Parameter | Value | Reference/Comments |
|---|---|---|
| Linear nucleator concentration [Nucleator] | {0, 10, 25, 50, 75, 100, 125, 150} nM {0, 25, 63, 126, 189, 252, 315, 378} molecules | Varied in this study |
| Nucleation rate $k_{bind}^{Nucleator}$ | 0.2 filaments/s | Arp2/3 nucleation rate [6] |
| Unbinding rate $k_{unbind}^{Nucleator}$ | 0.005 /s | Arp2/3 unbinding rate [7] |
| Actin elongation rate | $k_{grow}N_{fil}(1-\frac{[F-actin]}{[T-actin]})$ | [8] Changes with the amount of polymerized actin [F-actin] |
| Number of filaments $N_{fil}$ | 30 | |
| Total Actin | [T-actin]=138.38 $\mu M$ | Obtained by solving an ODE model as explained in Chandrasekaran et al. [9] |
| Filament extension rate ($k_{grow}$) | 0.0103 $\mu m/s$ | |

**Supplementary Methods**

1. <u>Simulation of VASP droplet with actin filaments in LAMMPS</u>

We perform molecular dynamics (MD) simulations of VASP droplets phase-separating in the presence of crowder molecules as elaborated by Cho *et al.*[1]. The simulations are performed in Lennard Jones (LJ) units. Simulations begin in a $60\sigma \times 60\sigma \times 60\sigma$ box with periodic boundary conditions. We consider a system with 10% by volume crowder (41,250 molecules) surrounding a condensed phase made of 20,625 VASP molecules. Condensed phase is initialized within a sphere of radius $60\sigma$. Both VASP and crowder molecules are modeled as spheres of radius $1\sigma$. The crowder molecule is used to mimic the PEG-driven demixing of VASP molecules as observed in experiments.
Crowder molecules interact through an LJ potential $U_{V-V}(r) = 4\epsilon_{V-V}[(\sigma/r)^{12} - (\sigma/r)^{6}]$, where $\epsilon_{V-V}$ is the interaction strength between two VASP molecules. To ensure that VASP molecules are attracted to one another, the LJ potential between VASP molecules is truncated at a radius of $2.5\sigma$. On the other hand, both the interaction between crowder-crowder ($U_{c-c}$) and crowder-VASP ($U_{C-V}$) are truncated at $2^{1/6}\sigma$ to ensure that the interactions remain purely repulsive. The system was simulated under N,V,T ensemble

under reduced temperature $T *= k_B T/\epsilon_{V-V} = 1.0$, where $k_B$ is Boltzmann constant and with time step $\delta t = 0.0005\tau_{MD}$, where $\tau_{MD} = \sigma(m/\epsilon_{V-V}\ \ )$. Actin filaments are represented as a series of beads of radius $0.1\sigma$ and length $10\pi$ (Half the cross-sectional circumference of the condensed phase). Beads are connected by springs and we also impose bending potential along the hinge points. Please refer to Supplementary Table 1 for details on LAMMPS[10] parameters used in this model. Droplets were equilibrated with actin filaments by adding 2 actin filaments at a time and equilibrating for $2 \times 10^6$ steps. Equations of motion are integrated using the leap frog algorithm and the simulations. Results corresponding to 6 and 20 filaments are discussed here (Supplementary Figure 1, B-D). To mimic VASP-driven crosslinking of actin filaments, we used the bond/create command in LAMMPS to allow for crosslinking of actin filaments. Crosslinking is attempted every 10,000 steps between binding sites separated by a distance $\leq 1.2\sigma$ with a binding probability of 0.5. These crosslinking events were permanent resulting in harmonic coupling of actin filaments.

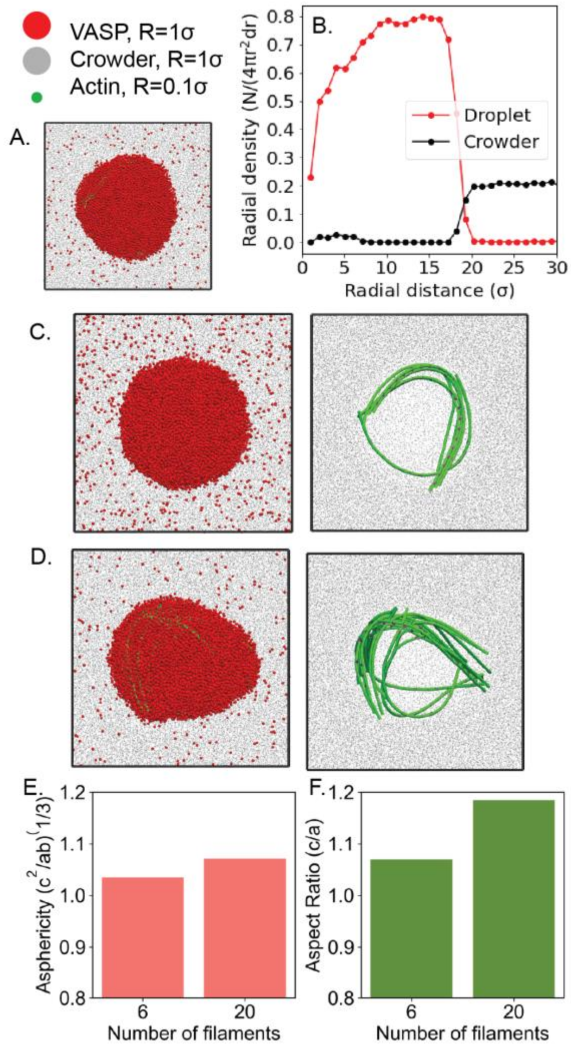


**Supplementary Figure 1. Detailed model for actin encapsulated droplet qualitatively matches continuum model predictions.** A. Droplet mimic consisting of VASP molecules (LJ), Crowder (visualized at a smaller particle size, colored gray) and actin filaments (shown in panels C and D as green filaments) are generated in LAMMPS. are Please refer to Supplemental Methods and Table 1 for detailed description of method and the parameters used. B. Radial density distribution of VASP (Droplet) and Crowder molecules. C. Final snapshot of droplet containing six actin filaments ($14 \times 10^7$ steps). Right subpanel shows corresponding actin configuration. Crosslinks between actin filaments are visualized as purple bonds. D. Final snapshot of droplet containing twenty actin filaments ($14 \times 10^7$ steps). Right subpanel shows corresponding actin configuration.  Crosslinks between actin filaments are visualized as purple bonds. E. Final asphericity index and F. Aspect ratio of droplet phase are plotted. Droplet interface was determined as the distance along radial density profile where density falls below 0.1. Source data are provided as a Source Data file.

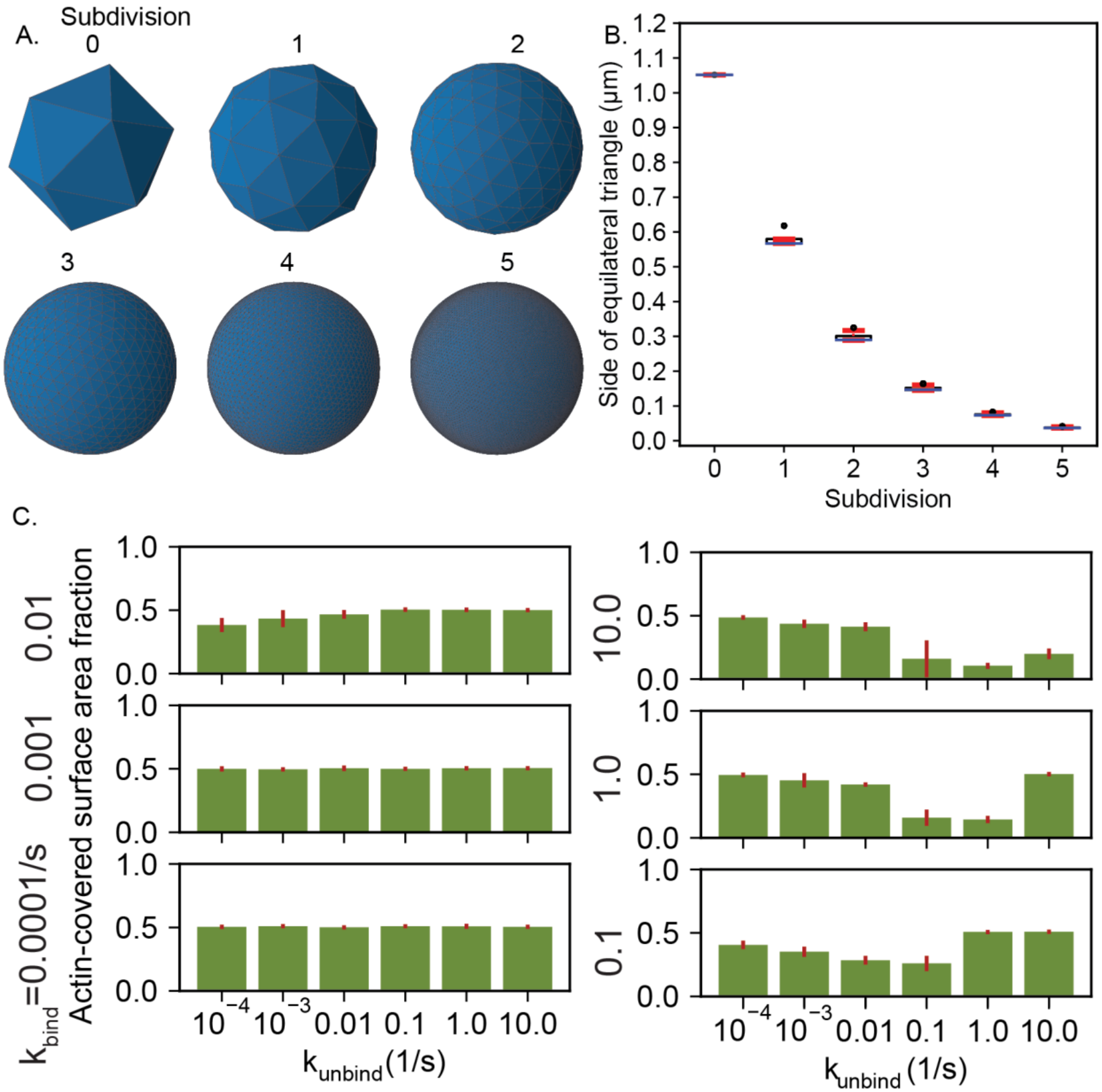


**Supplementary Figure 2. Steps used to obtain area fraction of actin.** A. Icospheres generated at various subdivision values. B. Box plot showing the distribution of the triangle side as a function of subdivision of the icosphere. Median (blue), quartiles (black), 95% confidence range (red) and outliers (black circles) are shown for each level of subdivision. C. Actin-covered surface area fraction is calculated by generating a surface actin density map as described in Supplementary Methods and shown in Figure 3. Bar graphs mean and error bars correspond to standard deviation of final actin-covered surface fraction under various unbinding and binding rates. Data from the last 30 frames from each of the three replicates were used. Source data are provided as a Source Data file.

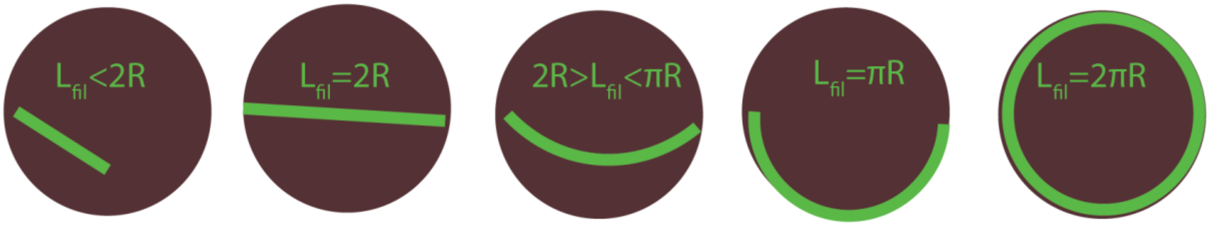


**Supplementary Figure 3.** Schematic description of the minimum-energy configurations within spherical droplet for actin filaments of various lengths.

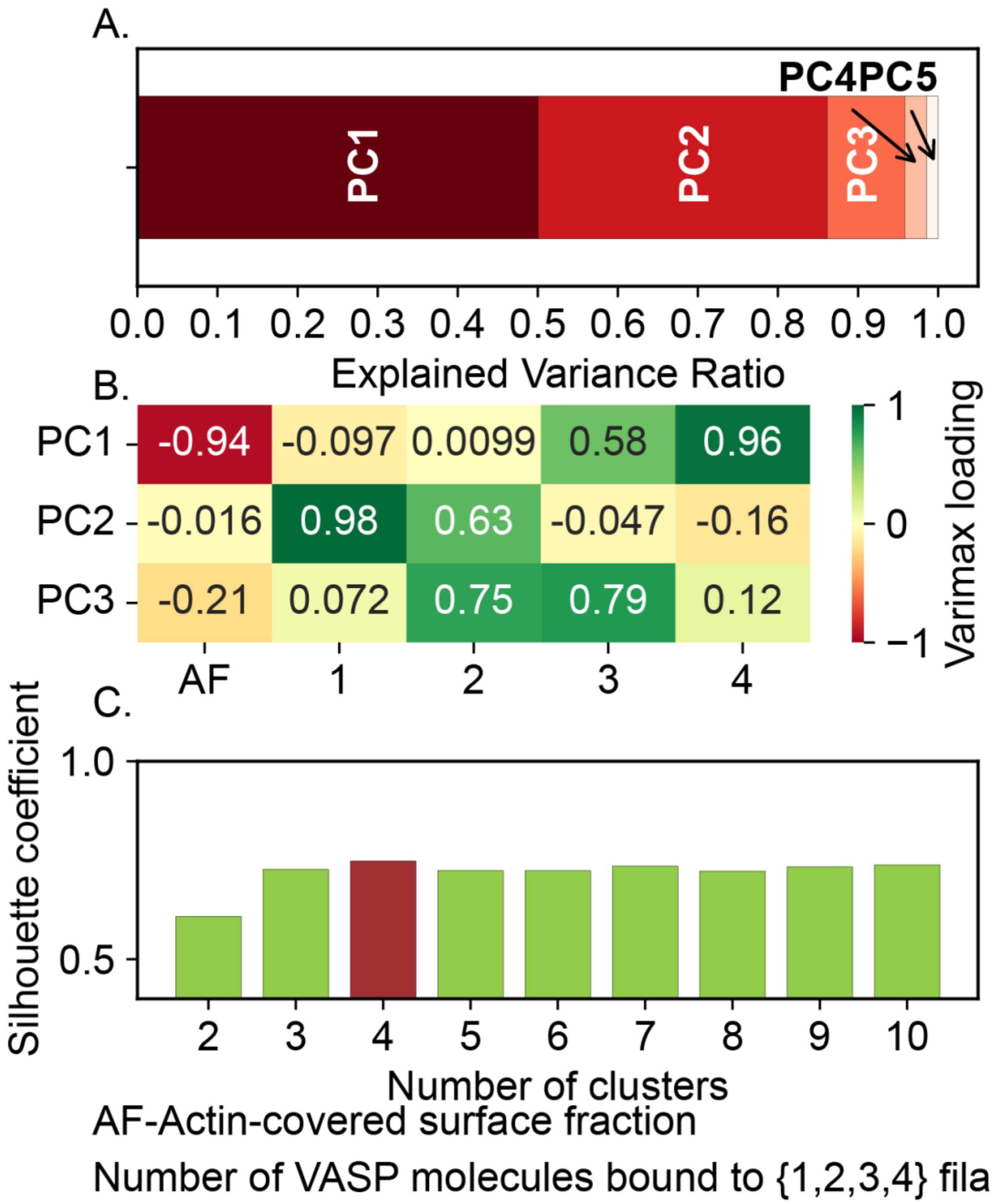


AF-Actin-covered surface fraction

Number of VASP molecules bound to {1,2,3,4} filaments

**Supplementary Figure 4: Feature and cluster number optimization to determine salient actin shapes present in our simulation.** A. Principal component analysis on the five order parameters shows that the first two PCs explain 95.83% of variance. B. Varimax loading of the first two PCs suggests that the first PC takes information of Actin-covered surface fraction, and fraction of VASP molecules bound to {3 and 4} filaments while the second PC is dominated by information from the fraction of VASP molecules bound to {1, 2} filaments respectively. C. Silhouette coefficient was calculated to find the optimal number of clusters in our dataset. Source data are provided as a Source Data file.

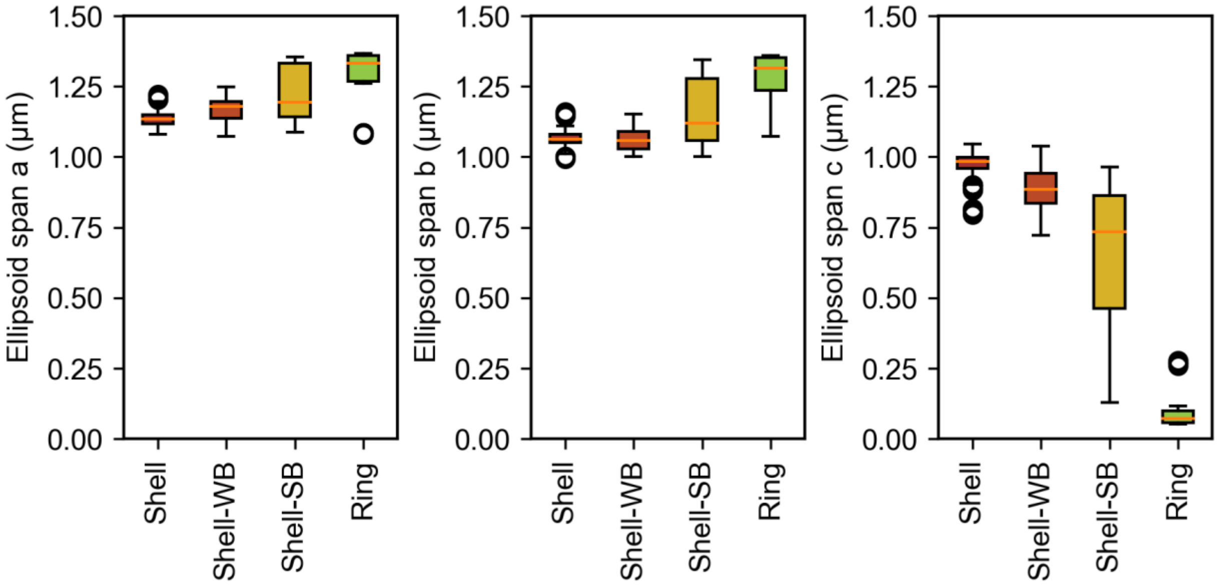


**Supplementary Figure 5. Ellipsoidal spans show differences between the four cluster categories.** Assuming the actin network shape to be ellipsoidal, the dataset shown in Figure 3E was analyzed. The resulting spans (a≥b≥c) are shown here as boxplots. The cluster category is mentioned along the X-axis (Shell-WB- Shell with weak bundling, Shell-SB - Shell with strong bundling). Median (orange line), quartiles (top, bottom edges of the box), 95% confidence interval (whiskers) and outliers (open circles) are shown. Source data are provided as a Source Data file.

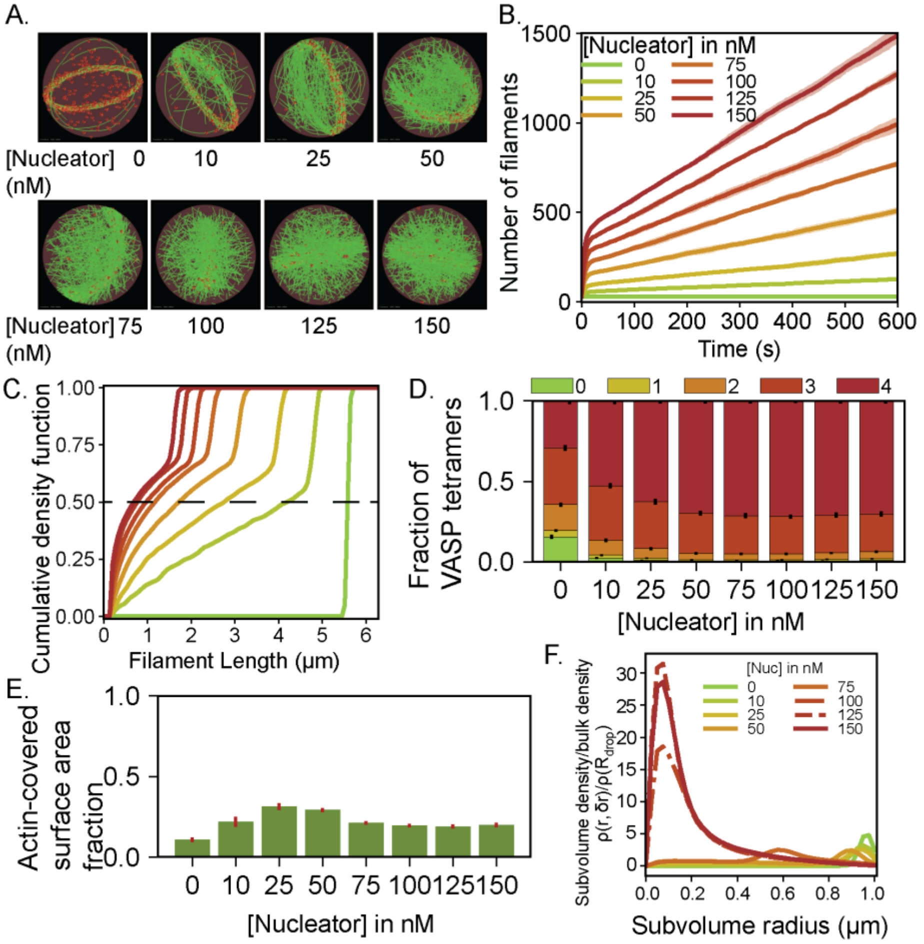


**Supplementary Figure 6. Nucleation causes changes to filament length distribution causing actin accumulation in the droplet core.** A. Representative final snapshots (t=600) from simulations at various linear nucleator concentrations. Actin filaments are shown in green while VASP tetramers are shown as red spheres. B. Time series of the number of filaments in the system at various nucleator concentrations. The mean is shown as solid line while standard deviation is shown as shaded area (5 replicates). C. The distribution of filament lengths at various nucleator concentrations are plotted as cumulative density functions. The median density is represented by the dotted line. D. Stacked bar graph shows the fraction of VASP tetramers that are free and bound to 1, 2, 3, and 4 filaments. Corresponding nucleator concentration is shown in X-axis. E. Mean surface area covered by actin is shown as a bar graph at various nucleator concentrations. Standard deviation is shown in red error bars. F. The droplet is divided into concentric shells of radius r, and thickness $\delta r = 25\ nm$. The ratio of density of actin within shells and the bulk density ($\rho(R_{drop})$) is plotted at various nucleator concentrations. B.,C, D., and E.. Data used: Last 30 snapshots from each of the 5 replicates per nucleator concentration. F. Data used: Last 10 snapshots from each of the 5 replicates per nucleator concentration. Source data are provided as a Source Data file.

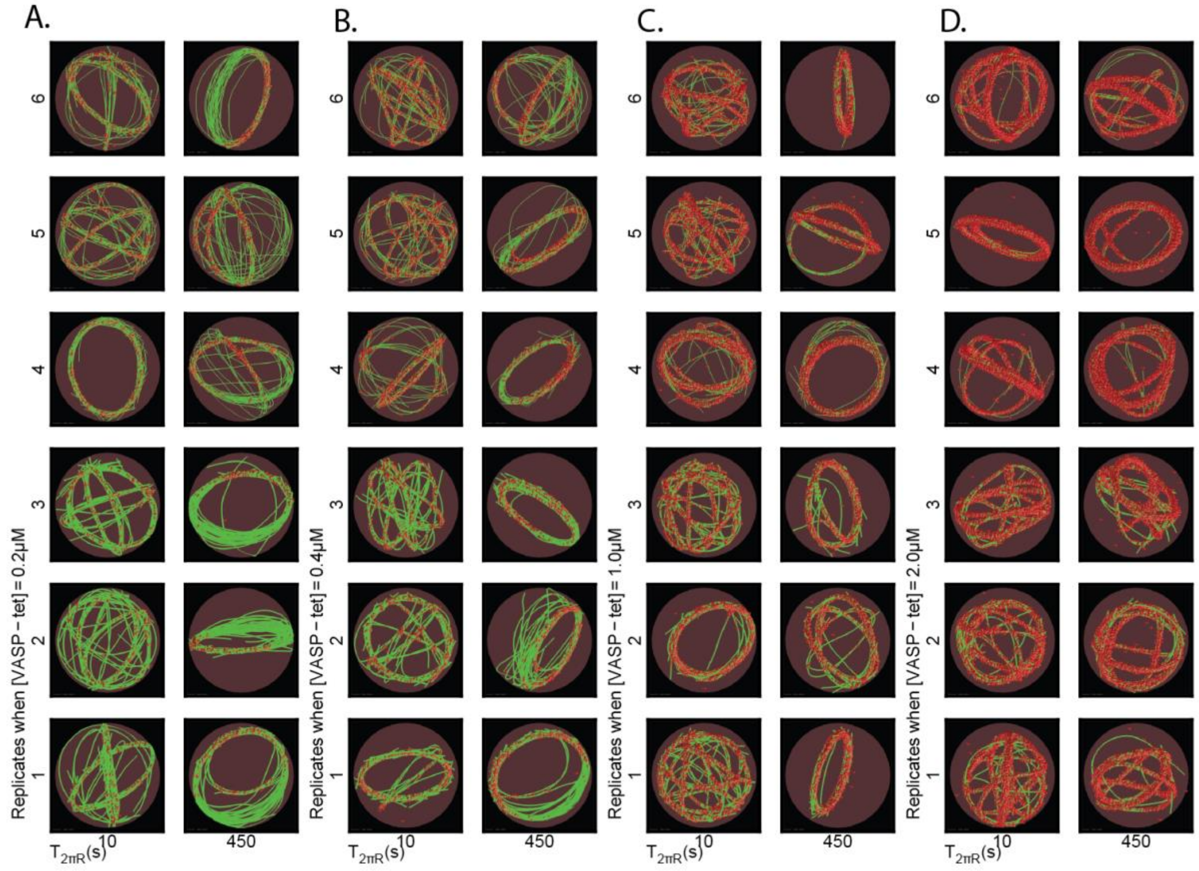


**Supplementary Figure 7. Heterogeneity in final network shapes at $T_{2\pi R}$=10s and 450s shown for tetramer concentrations.** A. 0.2 μM, B. 0.4 μM, C. 1.0 μM and D. 2.0 μM (5 replicates). Actin filaments are shown in green while VASP tetramers are shown as red spheres. $R_{drop}$ = 1 μm.

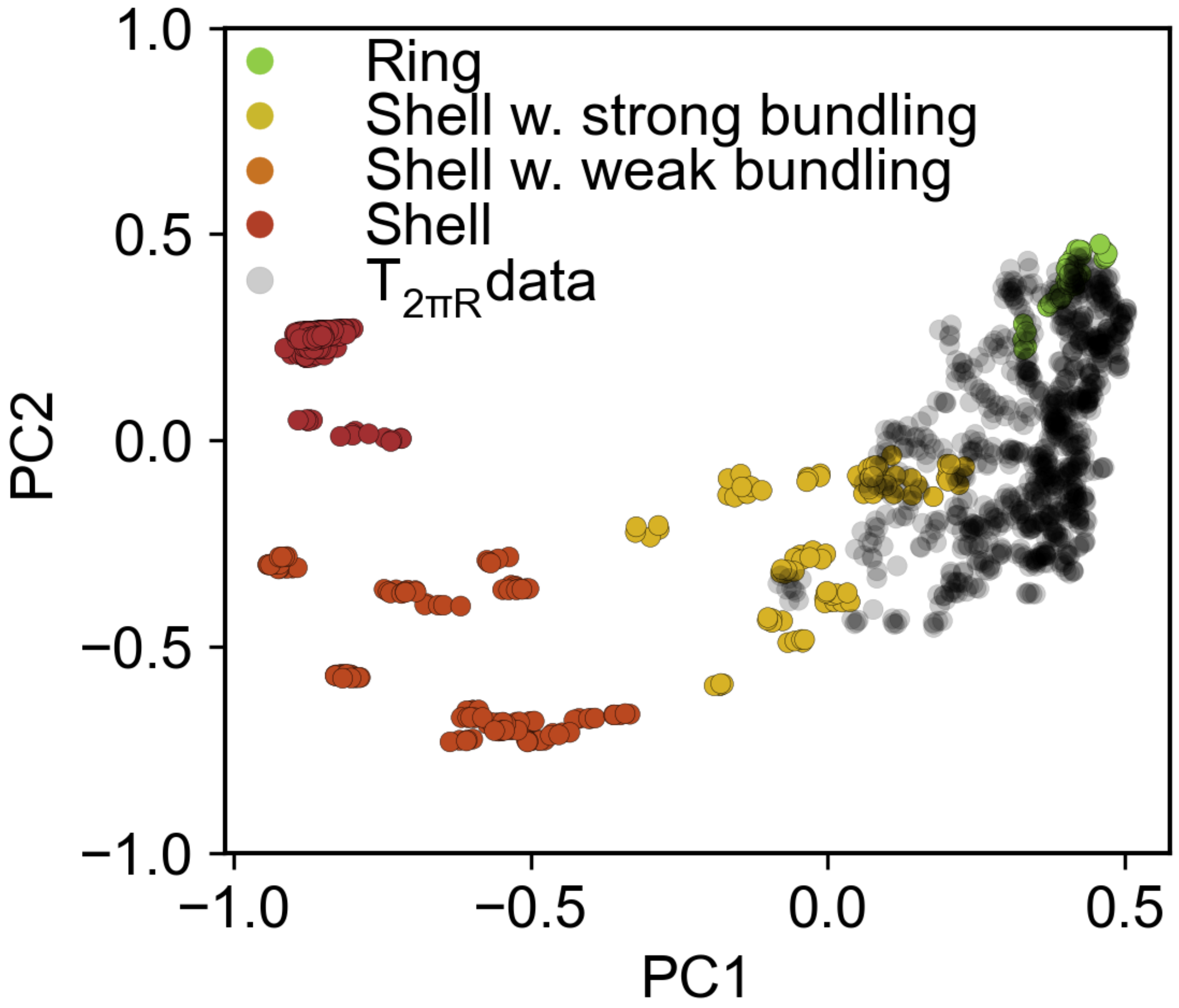


**Supplementary Figure 8. Data overlay highlights relevant shapes sampled by simulations at various $T_{2\pi R}$.** Data from simulations at various $k_{bind}$ and $k_{unbind}$ shown in Figure 2 (dataset 1) are combined with data from simulations at various $T_{2\pi R}$ shown in Figure 5 (dataset 2). The first two principal components are plotted. Data from dataset 1 are colored by their cluster identity while the data from dataset 2 are shown as black circles. Source data are provided as a Source Data file.

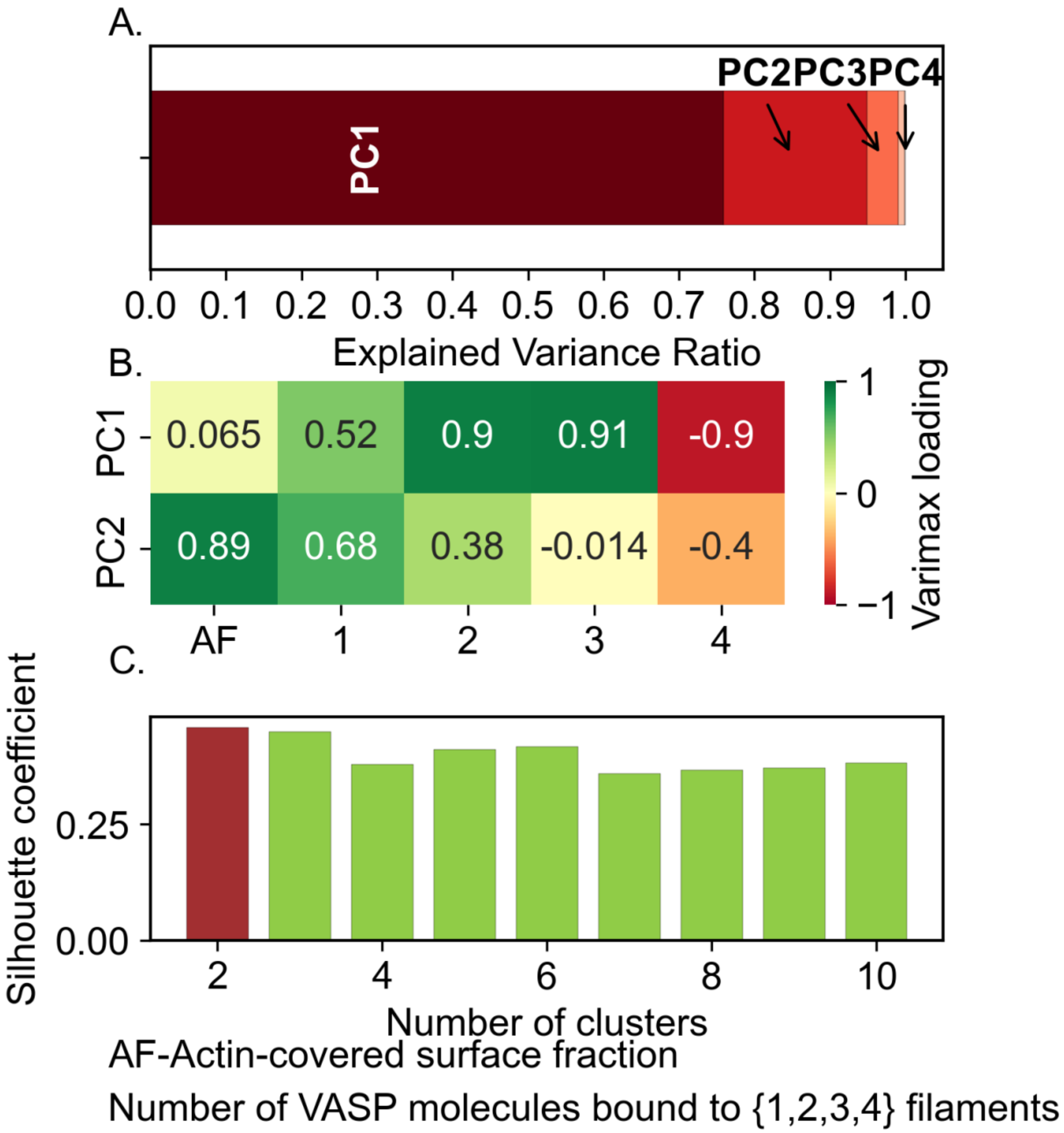


**Supplementary Figure 9. Feature and cluster number optimization to determine salient actin shapes present in our simulations at various $T_{2\pi R}$.** Data corresponding to rings, and rings with strong bundling from Figure 2 were combined with data from simulations at various $T_{2\pi R}$ (Figure 5). A. Principal component analysis on the five order parameters shows that the first two PCs explain 99.06% of variance. B. Varimax loading of the first two PCs suggests that the first PC takes information of fraction of VASP molecules bound to {2,3, and 4} filaments while the second PC is dominated by information of Actin-covered surface fraction, respectively. C. Silhouette coefficient was calculated to find the optimal number of clusters in our dataset. Source data are provided as a Source Data file.

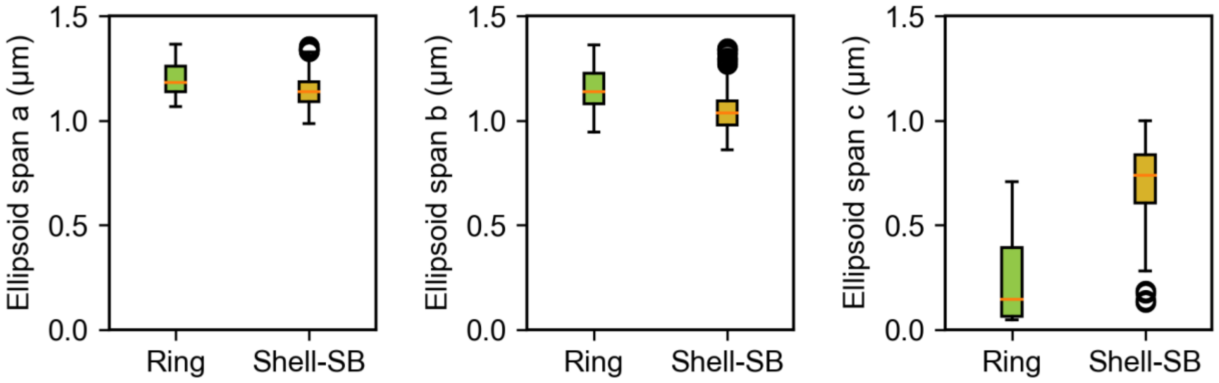


**Supplementary Figure 10. Ellipsoidal spans show differences between Ring and Shell-SB**. Assuming the actin network shape to be ellipsoidal, the dataset shown in Figure 5C was analyzed. The resulting spans (a≥b≥c) are shown here as boxplots. The cluster category is mentioned along the X-axis. Median (orange line), quartiles (top, bottom edges of the box), 95% confidence interval (whiskers) and outliers (open circles) are shown. Source data are provided as a Source Data file.

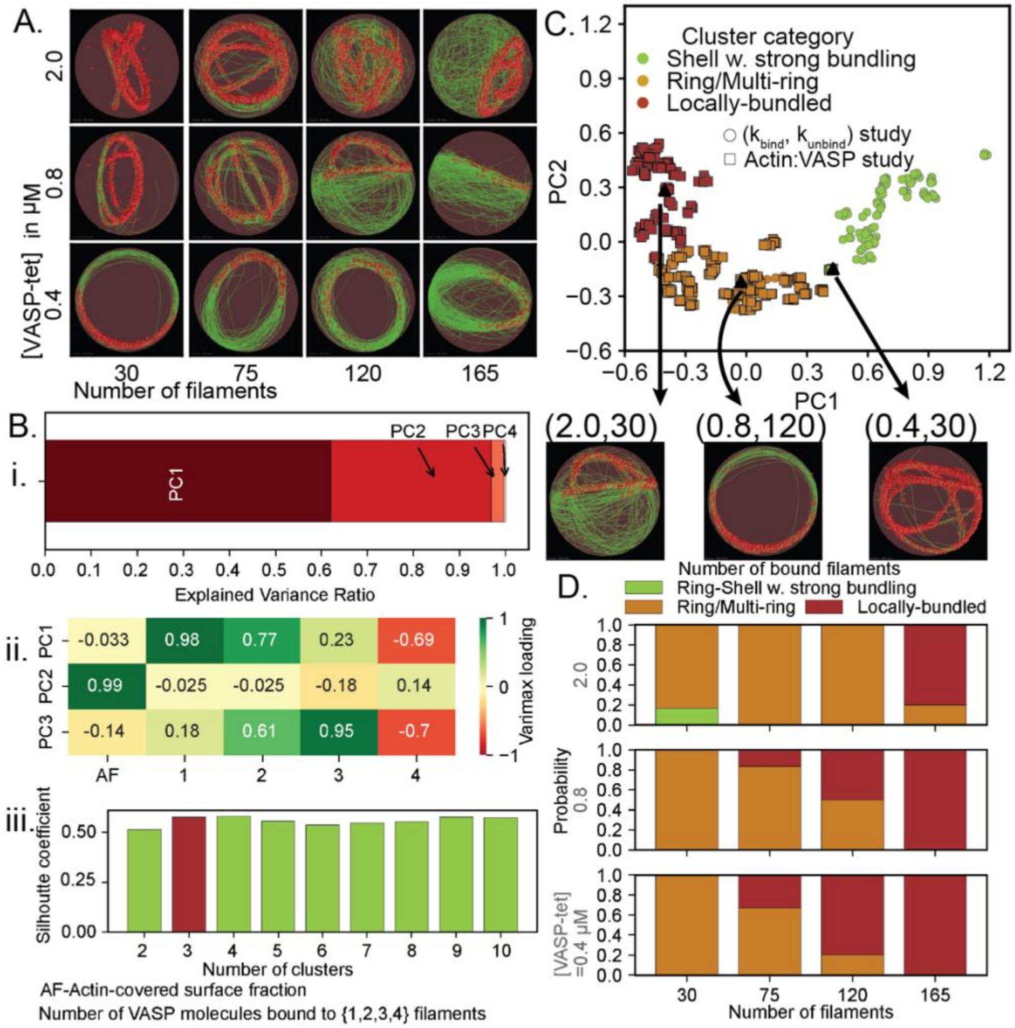

**Supplementary Figure 11. Networks with high actin concentration are characterized by diminished actin bundling.** A. Representative final snapshots (t=600s) from simulations at various number of filaments and [VASP-tet]. Filaments are shown in green while VASP tetramers are shown as red spheres. B. Feature and cluster optimization to determine salient network shapes. Dataset from rings, and rings with strong bundling from Figure 2 were combined with data from simulations in panel A (6 replicates). I. Two principal components explain 96.86% of variance in original data. Ii. Varimax loading shows that PCs 1, 2, and 3 take information from fraction of VASP bound to {1,2,4} filaments, Actin-covered surface area and VASP bound to 3 filaments respectively. Iii. Silhouette coefficient suggests using K-means clustering (with two PCs) shows the network has three clusters. C. Plot of first two PCs, where circles represent data points corresponding to rings and rings with strong bundling. Squares represent data from the last 30 snapshots (5%) from each of the 6 replicates shown in panel A. Data points are colored by cluster. Snapshot from dataset in panel A. closest to the centroid of each cluster is shown along with ([VASP-tet], Number of filaments). D. Probability of various network shapes is shown at various Number of filaments and [VASP-tet]. Source data are provided as a Source Data file.

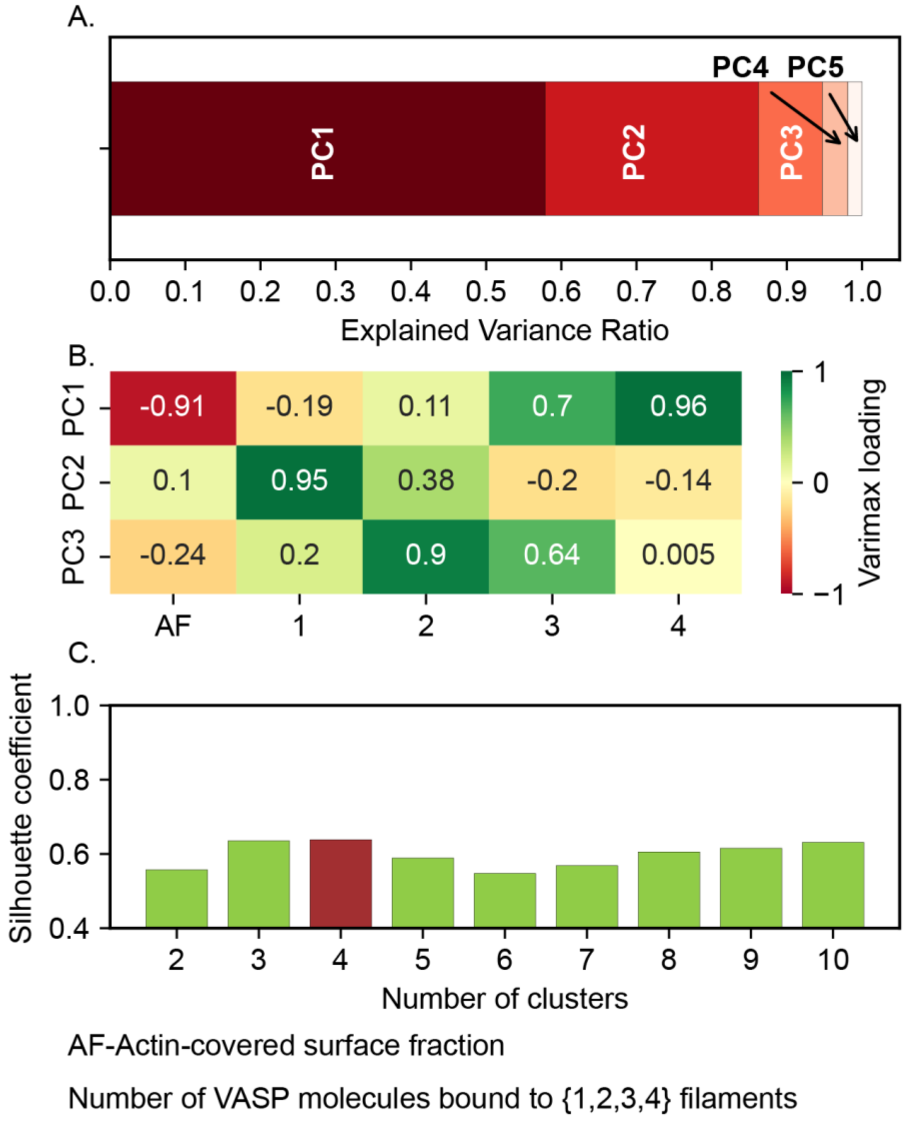


**Supplementary Figure 12. Feature and cluster number optimization to determine salient actin shapes present in our simulations with varying $k_{unbind}$.** A. Principal component analysis on the five order parameters shows that the first three PCs explain 94.72% of variance. B. Varimax loading of the first two PCs suggests that the first PC takes information of Actin-covered surface fraction, and fraction of VASP molecules bound to {3, and 4} filaments while the second PC is dominated by information from the fraction of VASP molecules bound to {1, and 2} filaments respectively. C. Silhouette coefficient was calculated to find the optimal number of clusters in our dataset. Source data are provided as a Source Data file.

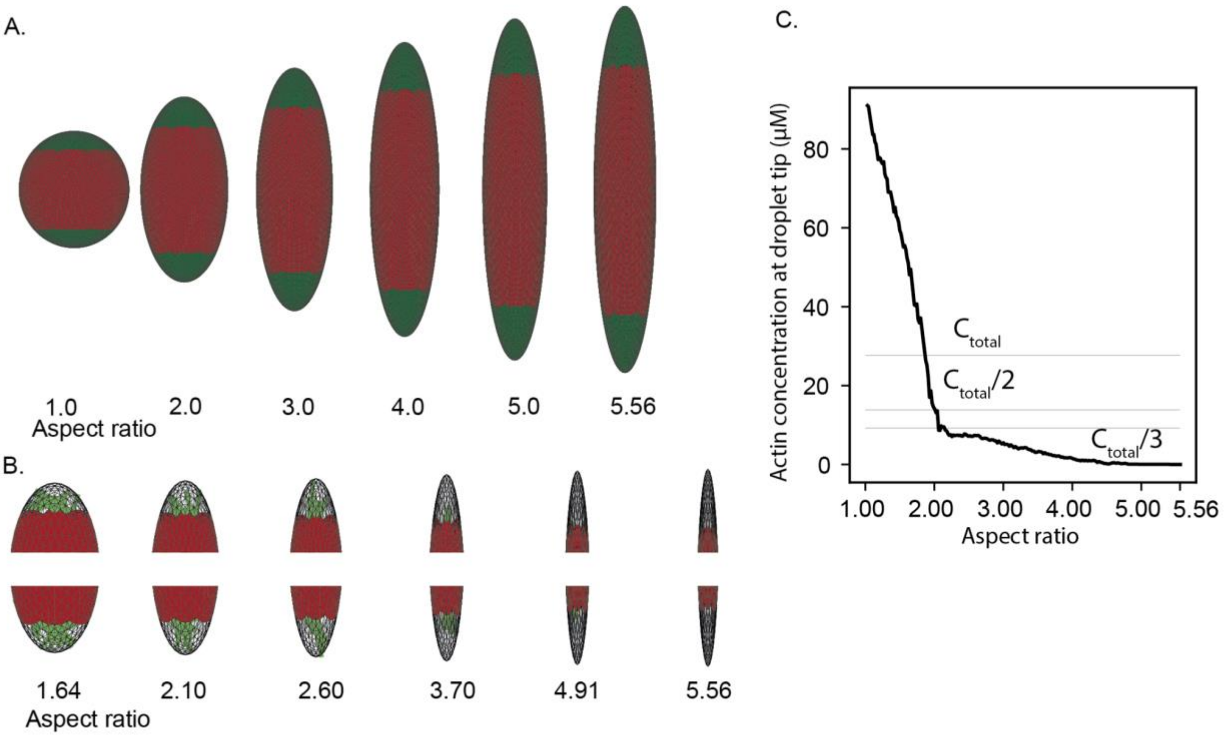


**Supplementary Figure 13. Determination of maximum aspect ratio.** A. Droplets of various aspect ratios are shown. B. The top and bottom caps of the droplet are shown at various aspect ratios from simulations where the Lfilmax is π μm. 12.5% (6.25% in the top and 6.25% in the bottom) of the droplet volume is shown as transparent subvolume along with actin filaments (green). C. The total actin concentration within the caps spanning 12.5% subvolume is shown at various aspect ratios for a representative trajectory ($L^{max}_{fil} = 13\pi R_{drop}/12$). Dotted lines represent 100%, 50%, and 33.34% of the total actin concentration within the droplet. The threshold of 33.34% and subvolume of 12.5% were chosen to identify the maximum aspect ratio of the deforming droplet. Above the maximum aspect ratio, we deem the deformation physically unrealistic. Source data are provided as a Source Data file.

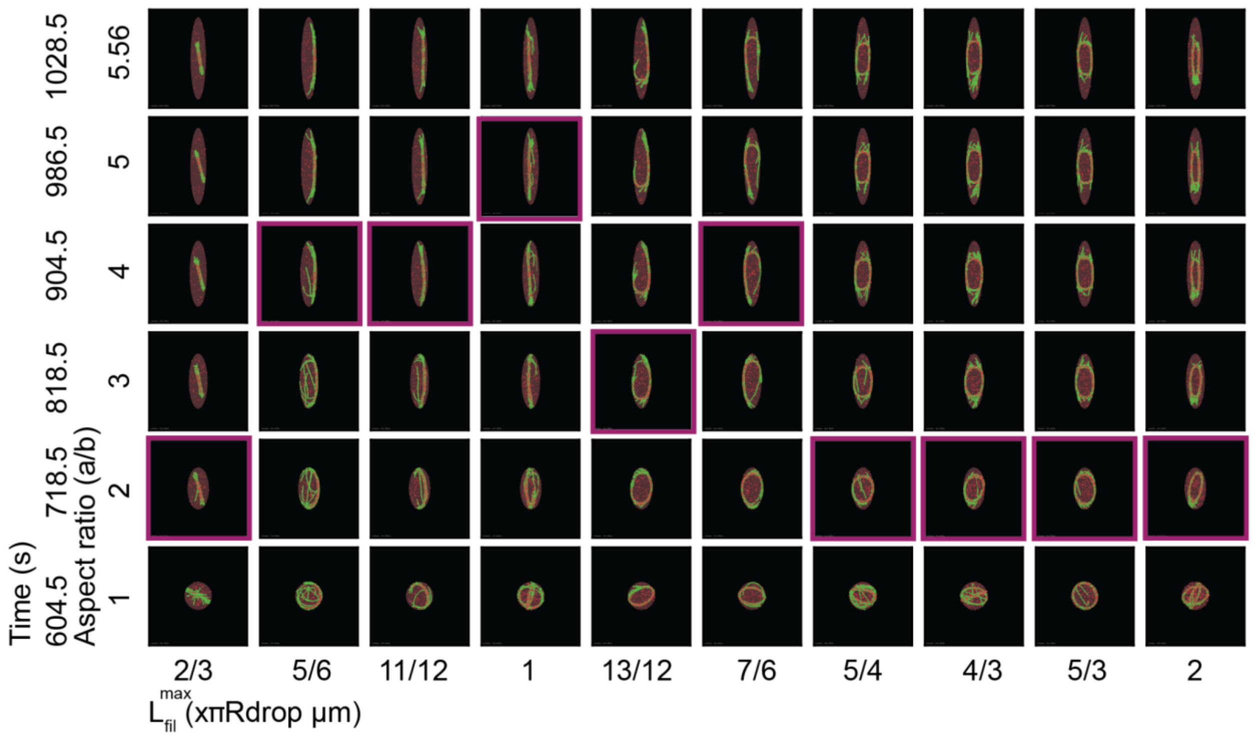


**Supplementary Figure 14. Snapshots from trajectories at various $L_{fil}^{max}$ are shown at specific time points along with the corresponding aspect ratios.** Snapshots with integral aspect ratio closest to the maximum aspect ratio are colored in purple. Each subpanel shows actin filaments (green), VASP-tetramers (red spheres), within the droplet volume shown in brown. The maximum aspect ratio was determined as mentioned in Supplementary Methods.

References

1. Cho, E. J. & Kim, J. S. Crowding effects on the formation and maintenance of nuclear bodies: Insights from molecular-dynamics simulations of simple spherical model particles. *Biophys. J.* **103**, 424–433 (2012).
2. Akenuwa, O. H. & Abel, S. M. Organization and dynamics of cross-linked actin filaments in confined environments. *Biophys. J.* **122**, 30–42 (2023).
3. Mogilner, a & Oster, G. Cell motility driven by actin polymerization. *Biophys. J.* **71**, 3030–3045 (1996).
4. Gittes, F., Mickey, B., Nettleton, J. & Howard, J. Flexural rigidity of microtubules and actin filaments measured from thermal fluctuations in shape. *J. Cell Biol.* **120**, 923–934 (1993).
5. Ferrer, J. M. *et al.* Measuring molecular rupture forces between single actin filaments and actin-binding proteins. *Proc. Natl. Acad. Sci. U. S. A.* **105**, 9221–9226 (2008).
6. Smith, B. A., Daugherty-Clarke, K., Goode, B. L. & Gelles, J. Pathway of actin filament branch formation by Arp2/3 complex revealed by single-molecule

imaging. *Proc. Natl. Acad. Sci. U. S. A.* **110**, 1285–1290 (2013).
7. Pandit, N. G. *et al.* Force and phosphate release from Arp2/3 complex promote dissociation of actin filament branches. *Proc. Natl. Acad. Sci. U. S. A.* **117**, 13519–13528 (2020).
8. Graham, K. *et al.* Liquid-like condensates mediate competition between actin branching and bundling. *Proc. Natl. Acad. Sci. U. S. A.* **121**, e2309152121 (2024).
9. Chandrasekaran, A. *et al.* Computational simulations reveal that Abl activity controls cohesiveness of actin networks in growth cones. *Mol. Biol. Cell* **33**, (2022).
10. Thompson, A. P. *et al.* LAMMPS - a flexible simulation tool for particle-based materials modeling at the atomic, meso, and continuum scales. *Comput. Phys. Commun.* **271**, 108171 (2022).
