# Supplementary material for: Kinetic trapping organizes actin filaments within liquid-like protein droplets
Source: Nat Commun. 2024 Apr 11;15:3139. doi: 10.1038/s41467-024-46726-6 (PMC11009352; doi:10.1038/s41467-024-46726-6)
Supplement: Supplementary file 2 — Reporting Summary [file 41467_2024_46726_MOESM2_ESM.pdf]

nature portfolio

Corresponding author(s): Padmini Rangamani, Jeanne C. Stachowiak

Last updated by author(s): Feb 26, 2024

Reporting Summary

Nature Portfolio wishes to improve the reproducibility of the work that we publish. This form provides structure for consistency and transparency in reporting. For further information on Nature Portfolio policies, see our Editorial Policies and the Editorial Policy Checklist.

Statistics

For all statistical analyses, confirm that the following items are present in the figure legend, table legend, main text, or Methods section.

n/a	Confirmed	
☐	☒	The exact sample size (*n*) for each experimental group/condition, given as a discrete number and unit of measurement
☐	☒	A statement on whether measurements were taken from distinct samples or whether the same sample was measured repeatedly
☐	☒	The statistical test(s) used AND whether they are one- or two-sided<br>*Only common tests should be described solely by name; describe more complex techniques in the Methods section.*
☒	☐	A description of all covariates tested
☒	☐	A description of any assumptions or corrections, such as tests of normality and adjustment for multiple comparisons
☐	☒	A full description of the statistical parameters including central tendency (e.g. means) or other basic estimates (e.g. regression coefficient) AND variation (e.g. standard deviation) or associated estimates of uncertainty (e.g. confidence intervals)
☒	☐	For null hypothesis testing, the test statistic (e.g. *F*, *t*, *r*) with confidence intervals, effect sizes, degrees of freedom and *P* value noted<br>*Give P values as exact values whenever suitable.*
☒	☐	For Bayesian analysis, information on the choice of priors and Markov chain Monte Carlo settings
☐	☒	For hierarchical and complex designs, identification of the appropriate level for tests and full reporting of outcomes
☐	☒	Estimates of effect sizes (e.g. Cohen's *d*, Pearson's *r*), indicating how they were calculated

Our web collection on statistics for biologists contains articles on many of the points above.

Software and code

Policy information about availability of computer code

Data collection	Simulation: https://gitlab.com/f-nedelec/cytosim<br>Experiment: All microscopy data was collected on a spinning disc operating on Olympus cellSens Dimension software v3.2
Data analysis	Experiment: ImageJ (v2.1.0) was used for image analysis.<br>Simulation: Python 3.9 scripts were analyzed using Jupyter notebooks.<br>The input files used, along with representative trajectories and data used to plot graphs will be made available on GitHub doi:10.5281/zenodo.10714332.

For manuscripts utilizing custom algorithms or software that are central to the research but not yet described in published literature, software must be made available to editors and reviewers. We strongly encourage code deposition in a community repository (e.g. GitHub). See the Nature Portfolio guidelines for submitting code & software for further information.

Data

Policy information about availability of data

All manuscripts must include a data availability statement. This statement should provide the following information, where applicable:

- Accession codes, unique identifiers, or web links for publicly available datasets
- A description of any restrictions on data availability
- For clinical datasets or third party data, please ensure that the statement adheres to our policy

The LAMMPS input files, initial and final configurations, along with CytoSim input files are available on GitHub doi:10.5281/zenodo.10714332. Source data for both experiments and simulations are provided with this paper.

Research involving human participants, their data, or biological material

Policy information about studies with human participants or human data. See also policy information about sex, gender (identity/presentation), and sexual orientation and race, ethnicity and racism.

Reporting on sex and gender	Not applicable as human participants are not part of the study.
Reporting on race, ethnicity, or other socially relevant groupings	Not applicable as human participants are not part of the study.
Population characteristics	Not applicable as human participants are not part of the study.
Recruitment	Not applicable as human participants are not part of the study.
Ethics oversight	Not applicable as human participants are not part of the study.

Note that full information on the approval of the study protocol must also be provided in the manuscript.

Field-specific reporting

Please select the one below that is the best fit for your research. If you are not sure, read the appropriate sections before making your selection.

☒ Life sciences ☐ Behavioural & social sciences ☐ Ecological, evolutionary & environmental sciences

For a reference copy of the document with all sections, see nature.com/documents/nr-reporting-summary-flat.pdf

Life sciences study design

All studies must disclose on these points even when the disclosure is negative.

Sample size	Sample sizes were chosen to yield sufficient data to model the trends and variability therein, and were also based on our previous experience (Graham, K. et al. Nat. Phys. 19, 574–585 (2023))
Data exclusions	No data was excluded.
Replication	All experiments were successfully performed and replicated at least 3 times using different stocks of protein to ensure biological independence and reproducibility.<br>All simulations were replicated at least three times. Exact replicate numbers are mentioned throughout the manuscript.
Randomization	Microscopy images were randomly acquired throughout the sample to avoid bias in data collection. Otherwise, randomization was not applicable as there were no assignments to experimental groups.<br>Stochastic simulations were performed in CytoSim with random seed generators.
Blinding	Blinding was not applicable, as no clinical or animal studies were performed. Data were quantified according to objective parameters as described in the methods. Microscopy images were taken randomly throughout the sample to avoid bias in data collection.

Reporting for specific materials, systems and methods

We require information from authors about some types of materials, experimental systems and methods used in many studies. Here, indicate whether each material, system or method listed is relevant to your study. If you are not sure if a list item applies to your research, read the appropriate section before selecting a response.

Materials & experimental systems

n/a	Involved in the study
x	Antibodies
x	Eukaryotic cell lines
x	Palaeontology and archaeology
x	Animals and other organisms
x	Clinical data
x	Dual use research of concern
x	Plants

Methods

n/a	Involved in the study
x	ChIP-seq
x	Flow cytometry
x	MRI-based neuroimaging
