# Supplementary material for: Kinetic trapping organizes actin filaments within liquid-like protein droplets
Source: Nat Commun. 2024 Apr 11;15:3139. doi: 10.1038/s41467-024-46726-6 (PMC11009352; doi:10.1038/s41467-024-46726-6)
Supplement: Supplementary file 4 — Description of Additional Supplementary Files [file 41467_2024_46726_MOESM4_ESM.pdf]

Title: Supplementary Movie 1.
Description: Movie showing representative trajectories showing actin filaments (green) growing (growth rate = 10.3nm/s) within R_{drop}=1um droplets of VASP-tetramers (red spheres) at various k_{bind} (mentioned on the left) and k_{unbind} (mentioned on the bottom) values . T_{sim}=600s, Δt_{frame} = 5s, $N_{filaments}$ = 30.

Title: Supplementary Movie 2.
Description: Movie showing representative trajectories of VASP tetramers (red spheres) simulated under the same actin addition rate but with different maximum filament lengths under ring forming conditions (k_{bind} = 10.0/s and k_{unbind} = 1.0/s) within R_{drop}=1um droplets. T_{sim}=600s, Δt_{frame} = 5s.

Title: Supplementary Movie 3.
Description: Gallery of representative trajectories showing actin filaments (green) growing at different filament extension rates specified by the time it takes for filaments to reach $2\pi R_{drop}$ (in seconds) at various [VASP-tet] concentrations within R_{drop}=1um droplets. VASP tetramers are shown as red spheres. T_{sim}=600s, Δt_{frame} = 5s, $N_{filaments}$ = 30.

Title: Supplementary Movie 4.
Description: Gallery of representative trajectories showing VASP tetramers (red spheres) simulated under the same actin addition rate but with different maximum filament lengths under ring forming conditions (k_{bind} = 0.1/s and k_{unbind} = 0.1/s). Actin filaments were simulated for T=600s within a spherical boundary (Video shows 2 snapshots from this part of the simulation) Later, the aspect ratio of the droplet was changed by 10nm each step (at constant volume) and the system was allowed to adapt for 1s before further deformation. Video sampled at 1 frame per second.
